# Methodological Considerations in Canine Olfactory Detection Research

**DOI:** 10.3389/fvets.2020.00408

**Published:** 2020-07-17

**Authors:** Lucia Lazarowski, Sarah Krichbaum, Lauryn E. DeGreeff, Alison Simon, Melissa Singletary, Craig Angle, L. Paul Waggoner

**Affiliations:** ^1^Canine Performance Sciences Program, College of Veterinary Medicine, Auburn University, Auburn, AL, United States; ^2^Department of Psychological Sciences, College of Liberal Arts, Auburn University, Auburn, AL, United States; ^3^Chemistry Division, U.S. Naval Research Laboratory, Washington, DC, United States; ^4^AGS Forensics, LLC, Washington, DC, United States; ^5^Department of Anatomy, Physiology, and Pharmacology, College of Veterinary Medicine, Auburn University, Auburn, AL, United States

**Keywords:** detection dogs, olfaction, scent detection, canine olfaction testing, animal behavior

## Abstract

Dogs are increasingly used in a wide range of detection tasks including explosives, narcotics, medical, and wildlife detection. Research on detection dog performance is important to understand olfactory capabilities, behavioral characteristics, improve training, expand deployment practices, and advance applied canine technologies. As such, it is important to understand the influence of specific variables on the quantification of detection dog performance such as test design, experimental controls, odor characteristics, and statistical analysis. Methods for testing canine scent detection vary influencing the outcome metrics of performance and the validity of results. Operators, management teams, policy makers, and law enforcement rely on scientific data to make decisions, design policies, and advance canine technologies. A lack of scientific information and standardized protocols in the detector dog industry adds difficulty and inaccuracies when making informed decisions about capability, vulnerability, and risk analysis. Therefore, the aim of this review is to highlight important methodological issues and expand on considerations for conducting scientifically valid detection dog research.

## Introduction

Dogs' superior olfactory abilities and high trainability are leveraged for a wide range of chemical and biological detection applications. As the scope of detection dog applications continues to grow, understanding detection dog olfactory capabilities and factors affecting performance is critical for improving training and deployment practices. However, methods for testing canine olfactory detection vary widely and such variation can influence the interpretation of results. Further, systematic reviews of canine olfactory detection literature have identified a major lack in reporting the information necessary to evaluate the validity of the results (1), as well as a prevalence of methodological confounds that could bias their interpretation (2). In contrast, analytical instrumentation undergoes rigorous validation standards prior to use in controlled and narrowly defined field operations. Here, we review the various critical features that should be included in the design and implementation of olfactory detection studies in order to ensure the quality and reproducibility of results. We expand upon the issues highlighted by Johnen et al. (2) to address considerations related to subject characteristics, experimental design, statistical analyses and reporting, and odor characteristics. Aspects of internal validity, or the extent to which results show evidence in support of what they claim, as well as external validity, referring to the ability of the results to generalize to populations, stimuli and environments other than those tested (3), will be discussed. The issues presented here should also be relevant for evaluating operational canine performance, for which there is also a lack of standardized protocols. While we argue for increased rigor in the examination of canine odor detection performance, the inherent variability of any biological system and technical challenges in its assessment across its wide operational field must be considered.

## Subject Characteristics

### Sensory Differences

Individual or breed-related differences in sensory and morphological traits can influence performance in olfactory detection tasks and thus the generalizability of results. These differences are especially important when examining performance capacities that are likely influenced by physical characteristics, such as threshold of sensitivity to low concentrations of odor. For example, genetic differences in olfactory receptor repertoire or anatomical variations in ear and nose shape may influence olfactory acuity (4). Bloodhounds possess 300 million odor receptor cells, more than any other breed (4), and are considered to have the greatest olfactory acuity (5). However, studies on canine olfactory threshold have been limited to single breeds (6–8) or few dogs from several breeds (9), with few studies examining breed as a factor in olfactory detection performance. In one study, differences in olfactory threshold were examined by comparing different breeds in a natural detection task in which food was hidden in containers of varying levels of permeability (10). Dogs from breeds selected for scenting abilities, such as hounds and beagles, exhibited a greater sensitivity than dogs from non-scenting breeds (e.g., grayhounds). Further, brachycephalic breeds showed the least sensitivity in detecting the odor compared to non-brachycephalic breeds. These findings suggest that differences in structure and function of the olfactory system may influence performance in an odor detection task, which could have implications for extrapolating results to other populations of dogs. However, whether the breed differences found in this study were specific to the nature of the target (i.e., raw meat) and would also be observed for a trained artificial target is unknown. Olfactory sensitivity and function is also influenced by a range of other factors including age, disease, medications, hydration, and diet [see (11) for review].

### Behavioral Differences

Contrary to evidence that breeding for scenting abilities and elongated noses is associated with better scent detection performance (10, 12), Hall et al. (13) found that pugs outperformed German shepherds in learning and performing a simple odor discrimination across decreasing concentrations of an odorant (13). This result is surprising considering the anatomical differences between these breeds and the popularity of German shepherds in scent detection work. Thus, these findings imply that other factors may influence performance on an odor detection task such as athleticism and behavioral differences, which may vary depending on the nature of the task. Indeed, German shepherds were not originally bred for odor detection tasks, but for herding and guarding sheep. Rather, the use of German shepherds for contemporary roles in the security sector is due to a combination of attributes such as athleticism, desire to work, and trainability necessary for multi-purpose work. Thus, had a more complex or strenuous task such as a search in an operational environment been used, German shepherds may have performed better than pugs in the Hall et al. (13) study. The importance of behavioral traits is further reflected by the fact that, despite their superior olfactory acuity, scent hounds are rarely used in olfactory detection research due to their poor trainability (14, 15). Other differences in olfactory search patterns, such as the tendency for nose-to-ground tracking vs. air-scenting, can influence performance depending on the type of detection task. An advantage of air-scenting is the ability to cover a wider range of search area in a shorter amount of time and to more efficiently locate targets using air currents (16). It is also important to recognize individual differences in motivation of the dog, as a lack of motivation to learn or complete a task could negatively influence the results. However, it is imperative to select an appropriate reward by using one with which the dog has experience or by conducting a reward preference test prior to the experiment, and to consider potential effects of reward value (i.e., highly preferred vs. less preferred) on performance (17). In addition, an easy warm-up trial prior to the session will ensure that the dog is willing to work for the chosen reward (18).

### Subject Selection

The sample of subjects selected may also influence the validity of the results obtained. In addition to differences in olfactory acuity or search behavior, training history can greatly influence detection performance. For example, experience with a particular odorant can affect sensitivity to that odor or generalization to other odors (5). Dogs specifically trained for scent detection are also more likely to perform better than novice dogs in search-based tasks. Thus, as acknowledged by the authors, the counterintuitive findings between pugs and German shepherds in the study by Hall et al. (13), which used privately owned pets, may have differed if purpose-bred or professionally trained detection dog German shepherds had been used. In applied research, it is sometimes imperative that the dogs used are representative of operational dogs for translation of the results to field applications. However, a potential concern when conducting research with operational detection dogs is that participation in the study could interfere with the dogs' operational performance. Recent studies have utilized privately owned pet dogs trained in sport detection (19, 20), which may represent a more practical model allowing for larger sample sizes and relevant experience. On the other hand, studies involving recruitment of pets may suffer from a sampling bias in which owners who volunteer their pets for behavioral studies may be more likely to engage their dogs in training and seek enriching activities. Similarly, studies using random source populations (e.g., shelter dogs) could introduce potential confounds related to the dog's experience, which is often unknown. Thus, the subject sample tested should always be taken into consideration when interpreting results, and efforts should be made to replicate and validate results in diverse populations and/or targeted to operationally relevant samples of dogs as laboratory derived results do not always directly correlate to the performance of operational teams as the subject population, behavioral requirements, target variables, environmental elements, and the canine handlers may not be the same.

## Experimental Design

### Sample Size

A further limitation related to sample selection is the number of dog subjects tested. Due to challenges in availability and access to dogs for extended periods of time needed for training and testing, the majority of dog studies utilize a small number of subjects. Although adequate for proof-of-concept experiments aimed at identifying a given capability, studies with few subjects complicate data analysis in more sophisticated experimental designs and limits the external validity of the results.

A recent examination of published dog studies evaluated the influence of sample size on effect sizes (i.e., the strength of the relationship between variables) and statistical power (i.e., the ability of the chosen statistical test to identify possible relationships between variables) (21), and found that the majority of dog studies were vastly underpowered and had low effect sizes due to low sample sizes (22). For example, the median number of subjects was 16, and the power produced by these studies was nearly zero. Statistically, a larger number of subjects allows for outcome-sensitive testing, meaning that the results are externally valid and highly replicable (22). Increasing the sample size is particularly important for group or matched-pairs designs due to variability between groups that could affect statistical power. When group designs are used, and especially when group sizes are small, all attempts should be made to equate the groups in terms of dog experience and capability. In cases in which increasing the sample size is impossible, researchers can maintain some level of external validity by increasing the number of trials and emphasizing individual differences (22). *A priori* power analyses can be used to determine the number of subjects needed in order to produce a desired effect size with narrow confidence intervals.

### Measuring Accuracy

A major goal of evaluating canine olfactory detection performance is to determine dogs' ability to correctly discriminate target odors from non-target odors. Accuracy of detection ability utilizes metrics utilized for medical diagnostics or analytical instruments, and is based on *sensitivity*, the probability of a response to a target odor when that target odor is present, and *specificity*, the proportion of non-targets correctly ignored (23). For example, studies of medical detection dogs' ability to detect certain diseases compare sensitivity and specificity between positive samples and controls, and can be used to compare dogs' performance to the best available gold standard for diagnostic technology [see (24) for review].

These metrics should be considered in tandem, as high sensitivity is meaningless if specificity is low (meaning dogs detect all targets, but also respond to non-targets), and a high level of specificity is not valuable if targets are also not detected. A low degree of sensitivity could be the difference between life and death for explosives or medical detection, and a low degree of specificity could lead to unnecessary response measures or anxiety (25, 26). A comprehensive assessment of canine olfactory detection accuracy then typically utilizes a signal detection theory approach, recording true positives (hits), true negatives (correct rejections), false positives (false alarms), and false negatives (misses) (27). The most commonly reported measures of performance include hit rates, calculated as the number of hits out of the total number of target exposures, and false alarm rate, calculated as the number of false alarms out of the total number of opportunities for a response (or conversely, correct rejection rate) (28, 29). Some metrics combine both sensitivity and specificity in order to measure overall accuracy, such as proportion of correct responses (hits and correction rejections) out of the sum of all responses (hits, correct rejections, false alarms, and misses) (30). A number of other metrics are also sometimes calculated depending on the measure of interest, such as positive predictive value (PPV) as a measure of how frequently a dog's alert is a correct one (20), false discovery rate (FDR; proportion of responses that are incorrect, or 1-PPV), and other variations.

### Types of Tasks

#### Discrete trials

Initial validation of odor recognition is typically measured as a dogs' ability to discriminate target odors from non-target odors (23). This is often achieved using controlled set-ups in which dogs are presented with a fixed number of positions to sample from which may contain targets or non-targets. Common testing arrangements include radial arm carousels or odor sampling arrays arranged in a circle or line, and dogs are trained to sample from each position in the array. Because dogs are presented with samples one at a time, these types of tasks are analogous to the “go/no go” task widely used in behavioral research in which an independent decision (yes/no) is required for each stimulus sampled. Thus, these types of tasks are considered discrete trial procedures because they consist of isolated opportunities to make a single response to a given stimulus, whether a target or non-target odor (31). Because of the precise control over the presentation of both targets and non-targets and subsequent responses (or lack of) to each, comprehensive performance metrics can be calculated. For example, in order to calculate true false alarm rate, the proportion of correct rejections of non-targets encountered must be known. In a discrete trials fixed sampling task, the number of positions containing non-targets that the dog checks and does not respond to prior to encountering the target can be counted.

Such procedures are common in canine olfactory detection research as well as in accreditation or proficiency testing of trained detection dogs, as they are easy to standardize allowing for comparisons across dogs or groups. For example, the Bureau of Alcohol, Tobacco, Firearms, and Explosives (ATF) developed the National Odor Recognition Test for proficiency testing of explosives detection dogs using a fixed-sampling circular arrangement (i.e., paint cans arranged in a circle) with defined testing parameters, thus allowing for a uniform assessment of dogs across varying agencies and organizations. A tool developed by Porritt et al. (23) automatically generates a test design with balanced order and number of trials, plus number and placement of distractors allowing for standardized comparisons and is available online for free to practitioners and researchers (23, 32).

A challenge to procedures requiring a decision response for each sample is the response inhibition required for a correct rejection. Olfactory go/no go studies in rats suggest there is significant difficulty learning to inhibit a response when presented with a non-target odor (33). Response inhibition is considered a form of self-control, which may be reduced in populations of working dogs bred for high energy levels and exhibiting higher levels of impulsivity (34). Researchers have suggested that refraining from making any response introduces needless difficulty which can be mitigated by training differential responding (2, 18, 33). For example, Edwards (28) trained dogs to hold their nose in a port emitting a target odor to indicate a “yes” response, and to remove the nose and push a lever for a rejection response (28). In multiple-choice arrays, a rejection response could simply be leaving the positions and moving on to the next one.

Another obstacle in using multiple-alternative arrays is the tendency for dogs to develop positional biases. For example, when the same positions are used and re-used within a training session, dogs have been shown to defer to responding to a particular position that was more recently or more frequently rewarded (35). This type of bias is more common early in training when the dog is not proficient in detecting the target, and should minimize as dogs' confidence in detecting the odor increases. Thus, it is best to begin training with few positions, increasing number of positions as dogs' proficiency increases, so as to reduce the cognitive demands of the task (14, 36). The position in which the target odor is placed should then be randomized so that patterns in placement that dogs' could learn are minimized. However, researchers have cautioned that a risk of full randomization is that for dogs already exhibiting a positional bias, randomization could lead to targets being placed in positions that the dog already preferred, thus reinforcing the positional bias (37). Thus, Jezierski et al. (37) suggested quasi-randomization in which the position of the target on each trial is tailored to the dogs' training deficiencies, which should be corrected before testing (with full randomization) begins (37). Another strategy for reducing positional biases is counterbalancing, in which each position contains the target an equal number of times across the session. An advantage of counterbalancing is that potential positional biases can be detected because each position is used equally, and therefore false responses or misses should be equally distributed across all positions unless a bias has developed. A common positional bias that has been reported is the tendency for dogs to emit a false alert in the last position of the array due to an increasing likelihood that a given position contains a target as the number of positions checked increases (2), or because the last position represents that final potential opportunity for reinforcement (32) [though the opposite pattern is observed in scent line-up tasks due to the memory component and increased delay the further away the targets are placed (14)]. Johnen et al. (2) suggest several strategies to remedy this last-position bias such as using a circular array with no discernible start or end point, and making the number of potential targets per trial variable and unpredictable, either by training dogs that an array can contain more than one target (and thus each position has a 50% chance of containing a target) or that it may contain no targets (i.e., blank trials) (2). Rewarding a correct rejection of a blank trial, for example by training dogs to perform a specific response if no targets are detected, can also reduce the tendency for false alerts (38).

#### Search Tasks

Discrete trial procedures may not be applicable to answering questions about operational search performance. Higher level and more complex skills related to search technique and ability are required during operational searches. Therefore, in order to assess detection dogs' ability to conduct a safe and systematic search in an operationally relevant manner as well as to validate initial odor discrimination testing, free searches are typically also employed (23). Free search tasks do not have defined opportunities for responses as in discrete trial procedures, but are used to assess other aspects of performance beyond basic odor recognition, such as dogs' ability to detect target odors in a complex environment in the absence of ostensive cues and independently follow the odor trail to its source. However, the increased complexity of the task makes evaluating performance in a free search scenario challenging in regard to standardization (2). One major limitation of evaluating performance during a free search is the infinite number of potential odor sources, and thus the inability to accurately calculate correct rejections needed to calculate true false alarm rates or specificity. Instead of calculating false alarms as a proportion of total opportunities for a response, one could calculate the proportion of false alarms as a proportion of the number of distractors placed in the search area. However, it is not guaranteed that the dog will necessarily encounter each distractor placed. Alternatively, PPV could be calculated which is the proportion of a dog's total responses (both hits and false alerts combined) that are correct. The higher the number of false alerts, the lower the PPV will be.

An additional layer of variability in dogs' performance in a free search task often results from handler error rather than errors by the dog. One commonly reported source of handler error is handler-induced false alerts (39, 40). Handlers may also cause dogs to miss targets by failing to ensure or inhibit the dog from adequately searching the area containing the target, making it difficult to differentiate whether a failure to alert to an odor (i.e., a miss) is due to a detection failure by the dog or handler error. For example, impatient handlers may rush the dog through a search (31), or may conduct an inadequate search pattern preventing the dog from having the opportunity to locate the target (41). In this case, it can be argued that the dog was not presented with the odor, which should not count as a true miss (though the distinction may not be as important for operational certifications, for example, where the handler performance is an important aspect of the team's ability). In cases where the detectability of the odor by the dog is of interest, researchers have addressed this challenge in a number of ways. Engeman et al. (41) utilized an inconspicuous observer to record whether the handler positioned the dog in a way that it was likely to detect planted targets in order to categorize missed targets as handler error or failure by the dog to give an alert (41). Another strategy by Porritt et al. (42) utilized “vigilance points,” in which specified locations unknown to handlers throughout a search area were identified to allow for data collection on whether or not the dog checked those locations (42).

There are several other ways in which handler factors can influence dogs' performance. Experience training and working with dogs as well as general familiarity with the dogs being tested can affect task acquisition (2) and interpretation of indication behaviors (12). Handler stress can also influence performance; for example, Jezierski et al. (12) reported dogs having longer detection times and more false alarms during formal certifications compared to informal examinations, which the authors attributed to handler anxiety due to the formality and pressure of the certification test (12). Schoon (18) reported that handlers' confidence was influenced by how the dog performed on a given trial, which could have influenced performance on subsequent trials (18). For example, if a handler calls an alert that turns out to be a false response, the handler may be hesitant to call subsequent responses by the dog. Dogs' performance has also been shown to be influenced by how familiar they are with the handler, with detection performance decreasing when working with an unfamiliar handler (43). On the other hand, Zubedat et al. (44) found that increased handler anxiety actually improved dogs' latency to detect targets (44). Interestingly, the authors suggested that handler stress led to a decrease in their control over the dog, thereby reducing handler influence and allowing dogs to work more independently. Further research is needed to directly examine handler effects, such as comparing dogs' performance when working on- and off-leash. Researchers should attempt to control for handler effects whenever possible by utilizing well-defined testing protocols, assessing both dog and handler performance to disentangle such variables, utilizing professional trainers, keeping the trainer/handler consistent, and keeping evaluators blind (discussed below).

Finally, detection of odors and search performance can be influenced by environmental factors such as temperature, humidity, air flow, and terrain (45). Hiding places for targets are also more variable in free searches, and odor availability can be influenced by depth, height, and containerization of the target odor. Placements of targets and non-targets should therefore be randomly distributed but matched in terms of level of difficulty or accessibility of target odors. Despite challenges in standardizing free searches, assessing performance in situations resembling real-world operations is critical for defining detection dog capabilities.

### Human Bias

#### Types of Bias

When a human handler or observer partakes in the administration or evaluation of canine testing, there are several potential sources of bias that can affect performance. One well-established form of bias in behavioral testing is known as the experimenter expectancy effect, in which observer expectations influence the subjects' behavior. This effect was famously illustrated by the classic example of the horse *Clever Hans*, believed to be capable of counting by stomping his foot a certain number of times in response to mathematical queries. In reality, the horse had learned to respond to unintentional cues by the people observing him, who exhibited subtle changes in body language and facial expressions as he approached the correct answer. Dogs are especially skilled at detecting subtle and unconscious cues given by humans. For example, dogs will often follow human cues that contradict available perceptual information (46, 47), and handler expectations about the presence or absence of a target odor can influence the team's accuracy (40). Allowing dogs to work off-leash can minimize handler influence, but does not remove all cues. Often unintentionally, handlers or observers may move more quickly past a search area known or expected to not contain any targets (19), or linger longer or pay greater attention in an area known to contain targets, which can provide strong cues to the dog regarding the probability of encountering a target. Edwards et al. (24) suggest that other unintentional cues given by experimenters or evaluators could influence performance as dogs are highly sensitive to human hand signals (48), body orientation (49), and emotional content of facial expressions and speech (24, 50). Methods for reducing observer influence in a search task have included positioning the observer on a designated mark on the floor, as well as requiring the observer to score whether or not the dog searched each target and non-target placed in the area so that the observer's attention to targets and non-targets was equal (23).

Even when care is taken to minimize potential cueing, knowledge or expectations held by the observer can influence the interpretation of the dog's behavior. Forms of observer bias are widely acknowledged in animal behavior research, such as selectively attending to information that confirms hypotheses or being susceptible toward certain beliefs based on prior knowledge. For example, observers scoring videos of animal behavior scored the same video differently depending on false information they were given about the animals or context of the video (51). Observer bias readily occurs when observers have a vested interest in the hypotheses or outcomes, when the behavior under observation is ambiguous, and when the interpretation of the behavior is subjective (51). Thus, there is risk of observer bias both in canine olfactory detection research in which investigators have expectations based on hypotheses, and in operational canine assessments when handlers and trainers have a vested interest in the dogs' success, and often occurs unintentionally.

Observer bias is inherent in canine testing due to the subjectivity of the behavior under observation. Dogs are trained to indicate an alert using a variety of responses such as sitting, lying down, and freezing, all of which require a certain degree of subjective interpretation. For example, whether a dog fully sat, and the duration of the sit, can lead to ambiguity in interpreting whether a response was made or not. Observer bias is particularly confounding when the response is ambiguous, as assumptions about whether or not the response is correct can influence its interpretation. Further, handlers may differ in how conservative their interpretation of a response is (28). This can complicate scoring when the dog's response is in conflict with the handler's interpretation; for example, a handler may believe a response to be incorrect and calls it a false, when the dog was actually correct. In this case, a decision has to be made whether to side with the handler or the dog. Whenever possible, an operational definition of a response defining the topography and duration of the behavior is critical, and should allow for different observers to come to the same conclusion (28). However, reliable agreement between observers does not necessarily eliminate observer bias if the two observers hold the same beliefs, as demonstrated by Tuyttens et al. (51) finding that the highest inter-rater reliability also had the highest degree of observer bias (51).

#### Minimizing Bias

Due to the many sources of human-derived bias in canine performance, blinding of personnel is critical. Single-blind testing is the most common procedure used in canine testing, where the handler is unaware of the test conditions (e.g., presence or location of targets) and an observer sets up the test problem and informs the handler of the outcome. This requires an ability to prevent visual identification of the target, as handlers can visually identify targets and voids the purpose of blinding. This type of protocol is often preferred by operational teams so that the handler can deliver timely feedback to the dog (e.g., reinforcement for a correct response) in order to maintain performance. Because the handler is unaware of the test conditions, handler influence on the dog's behavior is reduced. However, blinding the handler does not remove all sources of potential influence, especially when the evaluator is present and is not blind (52). The evaluator's behavior can provide strong cues as to the presence and location of target odors not only to the dog, but to the handler as well. In true operational situations, no one will know where the target is located. Thus, double-blind testing in which none of the participants or observers present in the test area are aware of the trial conditions (i.e., presence or location of targets) is the only assessment that truly reflects real-world operations (53). These situations also need to be mimicked in scientific studies whenever possible so the data can be directly correlated to operational performance, and in training to better prepare dogs for real-world scenarios. Indeed, studies reporting a decline in dogs' performance once double-blind testing is implemented underscore the importance of applying these procedures in research practice as well as in operations (54, 55).

A common solution to minimizing human influence is to position the handler and other observers in a way that they can view the dog but the dog cannot see them, such as behind a screen or one-way mirror (9). However, Edwards et al. (24) caution that removing visual cues is not always sufficient and other cues (e.g., auditory) may still be available (24). For example, the dog may learn to associate the sound of a pocket opening in anticipation of delivering a reward, or observers becoming quiet or holding their breath as the dog approaches the target location.

Double-blind testing is considered the gold standard in animal behavior research as it can minimize both observer bias and observer influence; however, double-blind testing is less commonly used due to challenges in its implementation. For example, one approach to double-blind canine testing is for both the handler and evaluator to be blind, and the handler calls out the dog's responses while the observer records the information. Once the trial is completed, someone who was not present during testing but knows the details of the scenario reviews the recorded responses and scores the dog's performance. This approach is often undesirable because accurate feedback to the dog's responses is not possible and non-differential reinforcement must be used (i.e., the outcome for correct and incorrect responses is the same). One option is to reward all of the dog's responses so that correct responses do not go unrewarded, but the risk is that false alarms can increase if incorrect responses are rewarded; the alternative is to withhold reinforcement of all responses, with the risk of performance or motivation declining (e.g., extinction). To prepare dogs for this type of testing, intermittent reinforcement schedules are often introduced in which reinforcement of correct responses is gradually faded so that some but not all correct responses are rewarded, resulting in behavior that is highly resistant to extinction (56). Accustoming dogs to intermittent reinforcement is especially important for preparing dogs for operational conditions in which reinforcing indications is not possible, such as in medical detection when the status of a sample is unknown (24). Another approach utilizing double-blind testing that allows for reinforcement of correct responses is for the blinded handler to announce when the dog makes a response, which is then confirmed by a third-party who is removed from the test situation (24). For example, in a study by Johnen et al. (1) the handler called out the number of the position where the dog responded, and then an experimenter out of view confirmed the response (1). This can be achieved by having the observer behind a screen or one way mirror, watching on a monitor connected to a video camera, or using a mobile device to communicate with the handler. Other systems have been used that do not require this type of relaying from one individual to another, such as custom-made software in which the handler presses a key to reveal the result (9). In all of these instances, a slight delay will be imposed between the dog's response and its reward, which can be introduced in training until the dog is accustomed to the delay.

To minimize subjectivity and increase the accuracy and reliability of testing, some researchers have devised automated approaches to data collection. For example, sensors that automatically detect a response by requiring breaking an infrared beam for a pre-determined amount of time reduce subjectivity in interpreting and recording whether a response has been made. Edwards (28) built a carousel apparatus for canine scent detection testing that automated all aspects of testing related to stimulus presentation, response recording, and reinforcement delivery (28). Infrared beams detected whether the dog observed a sample by requiring a minimum sniff time, ensuring that each sample was observed. Breaking the infrared beam for a longer pre-determined amount of time recorded an indication response, and correct responses were automatically reinforced via a feeder. Dogs were also trained to give a “no” response by pushing a lever which advanced the carousel to the next sample given that the minimum observation response criteria had been met, allowing for precise calculations of correct rejections. Though this type of system is more costly and requires significant training to teach the dog to operate making it impractical for operational assessments, research requiring precise control over stimuli and observations may benefit from automated systems such as this.

An argument to be made for non-automated response detection is that canine behavioral responses, especially in challenging or ambiguous situations, can sometimes be nuanced requiring a subjective but expertise-based interpretation. An example of this is a characteristic “change of behavior” (COB) interpreted by the handler as indicative of recognition of a target odor (57, 58). COB is often considered essential by handlers in declaring whether a dog has detected a target as the COB is considered a reflexive-like response to the conditioned odor that is not as encumbered by ancillary influences as the trained alert response (e.g., not being able to identify or access the exact odor source, or training deficiencies in performing the operant response). Because of this, a COB is often enough for the handler to declare a target. Further, in actual operations a COB is typically considered enough to prompt a threat response because waiting for a trained final response could be costly. Due to the high degree of subjectivity required in interpreting a COB, this metric should only be used when the handler is blind as observers are more likely to identify a COB when they are aware of the target location and not identify a COB in response to a non-target odor. Accordingly, COBs called to non-targets should be documented as a type of false response. For COB to be a meaningful measure of the dogs' response to a given target, the rate should be higher than COB to non-targets. Further research is needed to examine the specific behaviors accompanying a COB which may aid in standardizing the response as an acceptable metric as well as training observers to identify the response. For example, if the COB is truly a conditioned response elicited by a conditioned stimulus (the odor, which has been previously paired with an unconditioned stimulus), we may predict that the COB resembles behavior that is anticipatory of a reward, such as an orienting response (e.g., raised ears, looking toward stimulus) or approach (59).

### Odor Sample Controls

#### Positive Controls

Assessing canine olfactory detection performance requires constant scrutiny of extraneous variables by which olfactory behavior could be influenced. Positive controls are used to evaluate test validity, which, in the case of canine detection, ensure that dogs are responding to the target samples on the basis of the target odor. Positive controls involve the presentation of targets free from potential sources of contamination (60), such as new or refreshed samples, samples obtained from different sources (e.g., a different manufacturer or brand), preparation by a different person, or presenting the targets in new containers. A lack of responses to the positive controls suggests that dogs were responding to some other cue, such as contamination of the training sample that could occur from overuse, scent of the person that handled the odor, or the packaging material (2). Thus, positive controls are a necessary step for validating that dogs are capable of responding to the odor which they were trained to detect.

Positive controls are also useful to include during training to facilitate learning of the intended target. The use of a large number of positive controls has been shown to be especially critical in medical detection dog training as dogs have been shown to memorize the samples from individual people rather than the common odor (e.g., the disease). For example, Elliker et al. (55) found that the performance of dogs trained to detect cancer samples dropped when samples from new patients were introduced, indicating that the high accuracy observed to the training odors was due to memorization of the individual samples rather than the common odor profile (55). Training dogs to respond to odors based on some common classification essentially requires dogs to learn a concept, where the concept is the particular disease (e.g., cancer) or explosives class (e.g., chlorates) (24). Because dogs can readily learn and memorize a large number of individual odors within and across test sessions (61), it is recommended that training utilize a large set of samples. The larger the number of training samples, the more difficult it becomes to memorize individual samples and learning the concept common to all of the samples becomes a more efficient strategy. This “set-size effect” in which concept learning increases as a function of the number of training samples is a well-established phenomenon demonstrated in a range of species (62). The same principle applies to olfactory learning, where exclusively training with a particular odor or odor concentration tends to reduce the tendency to generalize to other variants or concentrations (63, 64). However, just as training with a fixed target can narrow the tendency to generalize to other variations of the target, training with a range of variants can enhance generalization (65). Research with detection dogs has demonstrated that the more that irrelevant factors are varied in training, such as source (66) and composition (29, 67–69), the more likely the dog is to generalize to other variants of the trained target. Thus, best practices for maximizing optimal generalization to potential variations of a trained target are to train with many exemplars of a target that vary by irrelevant dimensions (5, 70).

#### Negative Controls

Distractor odors used as *negative controls*, also referred to as interferents (23), consist of non-target odors and are equally critical in evaluating dogs' detection performance in terms of calculating specificity. The use of distractors is also important during training for teaching dogs to discriminate the target odors from non-target odors (e.g., discrimination training). For example, Elliker et al. (55) speculated that early training with only the target odors may have biased dogs toward memorization of the samples, and suggests that teaching dogs to disregard the controls by never presenting the target samples alone may be a better approach (55). Distractors should include odors that are similar to the target in terms of intensity, otherwise dogs could learn to differentiate the target odor based on its relative (higher or lower) strength, and should include odors from similar and differing odor categories (e.g., chemical, biological). Distractors should consist of odors commonly associated with training, the training environment (e.g., reward odors, handler/trainer odors), target containers (e.g., nylon bags), and preparation (e.g., gloves, pipettes), as these odors are likely to become associated with the training odor. For example, a preliminary study found that dogs trained to low levels of an explosive were actually detecting residue plasticizers from the pipettes used in preparation procedures (71). During search tests there are likely to be a variety of items present in the environment, but these items are likely acclimated to the environment which the dog may learn to ignore. Thus, other items should also be added and trainers and observers should touch various items in the area in order to introduce odors associated with human activity/disturbance similar to the disturbance that will be created by planting the target odor (72).

Items that systematically covary with the presentation of the target itself should also be presented as distractors. Sometimes referred to as *matched controls* or *matched blanks*, these distractors are designed to match the background odors that coincide with the target sample (73). In substance detection, matched controls should consist of empty and clean packaging materials and containers identical to those used to store or present the target odor, as well as gloves used to prepare and handle targets. In medical detection, matched controls consist of samples from patients of the same age and sex, as well as samples from patients with ailments different than the target disease but affecting the same organ so that samples are as comparable as possible and only differ by the specific disease status, eliminating other factors that covary with the disease (24). The use of matched controls during training helps dogs isolate the target odor, and during testing ensures dogs are responding to the target odor only.

Distractors should also include novel odors, particularly when testing for generalization to target odors that were not used in training. This is important because in such testing, the target odors will be novel to the dog. In order to ensure that any responses to the test odors are due to generalization based on the target odor and not due to responding to the anomalous odor, other odors that are novel should be present. Because dogs tend to be neophilic (74), disruption of performance during testing can be prevented with adequate discrimination training in which novel distractors are introduced early in training and are gradually faded in so that dogs learn that novel odors may be present at any time but do not learn any value associated with them (57).

Care should also be taken to remove visual cues that dogs could use to potentially identify targets. Although olfaction has been shown to be the dominant sense used by trained detection dogs to locate targets when compared to vision (75, 76), other studies have shown that in some contexts, such as when a human gesture conflicts with an olfactory cue, dogs may defer to visual cues (46, 47). Further, the use of distractors and controls requires a systematic approach of managing the materials, which often involves visually marking the materials. Dogs have dichromatic vision, expressing only two forms of light-sensitive photo pigments in the cells of the retina pertaining to color as compared to humans which express three forms and are trichromatic. Though this is generally considered to result in dogs exhibiting deutreranopia, a human-like red/green color blindness (77, 78), studies have demonstrated that dogs are capable of discriminating colors based on differences of brightness intensities (79, 80). Although color is thought to be predominant over brightness in canine visual processing, caution must be used if utilizing color coding in sample management as the colors may still be perceived differentially and could result in a visual cue being associated with the target.

### Criteria Testing

Before formal testing occurs, it is important to validate training and establish that dogs are prepared for testing. For example, when testing whether dogs generalize from a trained odor to an untrained odor or whether a dog will be successful at detecting a trained odor in a different context, researchers often require that dogs meet some pre-determined performance criteria [e.g., (81, 82)]. The criteria often consist of a minimum hit rate to the trained target odor and a maximum false alarm rate. For example, Porritt et al. (23) developed a pass criterion based on signal detection theory in conjunction with subject matter experts, resulting in an acceptable pass criterion of at least a 70% higher hit rate than the false alarm rate. The direct comparison between hit and false rate requires that individual dogs respond to their trained target significantly more often than they commit a false alarm in order to meet the criteria (23). If dogs' ability to meet a performance criterion prior to testing is not demonstrated, test performance will be unclear. Furthermore, conducting criterion tests with all controls in place provides a baseline measure of performance and provides dogs experience with the experimental design that will occur in testing so that performance is not disrupted when test protocols are implemented.

Acceptable accuracy rates vary across researchers and organizations, and should be pre-determined based on the goals of the testing. Ideally, a training criterion should enable researchers to be confident that the dog is prepared for testing and allow meaningful comparisons to test performance (discussed below). More stringent criteria may be required for explosives detection dogs being trained for operational deployment with greater risks associated with errors, or for drug detection or forensics dogs for which training records may serve as probable cause or evidence in court. It has been argued that true detection accuracy should approach 100%, but such expectations may be unreasonable considering the variety of factors related to odor presentation, odor source, and other test parameters (12). In some circumstances, purposefully tailoring training toward a liberal bias in responding when target odor is present (e.g., aviation explosives detection) or toward a conservative bias in not responding when target odor is absent (e.g., drug detection) is warranted.

### Test Parameters

In order to ensure validity of the results, specific session and trial parameters should be considered when evaluating performance. Most notably, both target and blank trials (i.e., no target odor present) should be included and should be randomized across the test session. In addition to reducing false alerts and positional biases as discussed above, blank trials are useful in keeping the probability of encountering a target on each search unpredictable to both the dog and handler. For example, if a target is placed in position five on a six-position wheel, every time a dog samples an empty position there is an increasing chance that the next position contains a target. Thus, detection rates for targets in later positions could be artificially inflated. By inserting blank trials, the dog cannot determine if a later position is likely to contain a target or if it is a blank (2), and the handler will be unaware of whether a lack of response was a miss or a blank trial. As mentioned above, varying the number of targets present on each trial is an alternative to inserting blank runs, though is arguably less practical.

This design is sufficient when dogs are trained to sample systematically and are quite accurate, however, adjustments are sometimes required. For example, it may be necessary to allow dogs to rerun the trial or search if an area is missed, the dog displays a COB, or shows interest but doesn't respond (9). Critically, the decision of whether the dog sufficiently searched the area or not should be made by a blind handler or evaluator before any feedback of the trial outcome is given. It is also important to note that allowing a dog to resample positions or re-run trials complicates calculating the correct rejection rate, and thus a priori decisions should be made regarding which run will be counted toward data analysis.

The number of test trials performed is another important consideration and should be determined based on statistical validity. As discussed above, statistical power will be influenced by the number of subjects which can be determined by an a priori power analysis. When the number of subjects is difficult to control, a priori analysis can also be used to determine number of test trials to determine a specific effect (24). However, the effect of repeating test trials for an individual subject should be considered. For example, rapid within-session learning can occur after repeated exposures if responses on test trials are reinforced (83). Alternatively, withholding reinforcement for responses on test trials can lead to within-session extinction. One option to reduce learning or extinction across trials is to implement intermittent reinforcement prior to testing so that performance is maintained in the absence of reinforcement, or to non-differentially reinforce correct and incorrect responses (29). Controlling for within-session changes in responding is especially critical in generalization studies when the goal is to assess spontaneous responses to an untrained odor, given that dogs are capable of learning to respond to a new odor in as little as 2–3 exposures (81). Thus when possible, the number of test trials should be limited in order to give a more accurate representation of initial response to the odor. When sample sizes are low and repeated test trials are needed to obtain sufficient data, first-trial performance or changes in responding across multiple exposures to the test odor should always be analyzed.

Within-session changes in motivation can also occur if testing is too difficult or too many non-reinforced trials occur. In order to maintain motivation during these testing sessions, reinforced baseline target trials are often dispersed throughout the session or search (24, 84). The inclusion of baseline trials during a test session also allows for a comparison between hit rates on baseline and test odors. For example, in generalization testing, comparing responses to trained and untrained targets is necessary for determining whether generalization occurred (85). Specifically, if the number of hits to the test odors is not significantly different than the number of hits to the trained target than it can be concluded that the dogs successfully generalized. In addition, comparing hit rate on test odors to hit rate on non-target odors as well as to random chance allows for an assessment of the degree of generalization. Responses to test odors that are significantly below baseline hit rate, but significantly above chance, could indicate that some degree of generalization occurred. Responses to test odors that are not significantly different from the false alarm rate indicates a lack of specificity which likely inflated hit rate. Borrowing from studies of animal concept learning, generalization that is equivalent to baseline and significantly above random chance could be considered *full transfer*, generalization that is below baseline but above chance could be considered *partial transfer*, and generalization that is not statistically different from chance could be considered a failure to transfer (62).

## Considerations Related To ODOR

### Characterization of Odor Samples

In order to properly conduct olfactory detection research, it is imperative to have a clear understanding of the odorants that make up the odor of the substance to be detected and approximately how much of it is being presented during olfactory testing. Without an understanding of the odor being delivered, one risks testing or training the dog on a set of odorants or quantity of odorants different than intended. There are several factors to consider regarding odor characterization, perception, and availability, discussed in the following section.

#### Qualitative Characterization of Odor

Generally, if a target material is not in the gas phase, it cannot be detected through olfaction (it is possible that dogs are capable of detecting very small particles that enter the nasal passageway, but should this be the case, odorant molecules on the particle are likely volatilized in the nasal cavity and ultimately detected in the gas phase, or broken down within the mucous layer and delivered to the olfactory receptors by transport proteins). Often times the molecules making up the target material are too large to be readily available in the gas phase. Instead, the animal will detect an associated odorant or collection of odorants that are unique to that target. These odorants are often referred to as the active odor (odorants) (60), and have been studied for many substances relevant to canine detection (86–93). This is of particular importance when considering detection of a target material with a very low vapor pressure, such as many narcotics or explosives. For example, cocaine is a large molecule with an accordingly low vapor pressure [303 g/mol; 3 x 10^−7^ Torr at 20°C (94)], and is not readily available in the vapor phase. However, methyl benzoate, a degradation product of cocaine is smaller in size (136 g/mol) with a higher vapor pressure (3 x 10^−3^ Torr at 20°C). In regard to testing detection thresholds of a low volatility substance such as cocaine, it is imperative to understand that this threshold is related to the amount of methyl benzoate present and not the amount of solid cocaine.

In most circumstances, the recognizable odor of a target material is not made up of a single odorant, but of a mixture of odorants, referred to as the odor profile. For instance, cadaver odor consists of hundreds of individual volatile and semi-volatile analytes that together create a unique odor profile recognizable by trained human remains search dogs (95, 96). For complex odor profiles such as living human and cadaver scent, it can be quite difficult to delineate which compounds the dog uses for detection, or if extraneous odors from contamination have added to or altered the odor profile.

#### Quantitative Characterization of Odor

The quantity of odor available is as equal a concern as the quality. Returning to the cocaine example, research has also shown that the quantity of methyl benzoate present from cocaine is dependent on the type of cocaine with pharmaceutical-grade cocaine yielding a significantly lower amount of methyl benzoate than that from street cocaine (88). Furthermore, research with ammonium nitrate, another low volatility substance, has shown that variations in the source and purity of ammonium nitrate as well as in the amount of ammonia influences the detection of ammonium nitrate (85, 97), demonstrating the importance of being mindful of possible variations in odorant concentration between related substances.

There is a common misconception that the amount of odor available can be easily altered by increasing the mass (or volume, in the case of a liquid) of the material (i.e., 10 g of a given material will yield 10 times as much odor as 1 g of the same material) (98). While mass or volume of a given substance is correlated to odor availability, increasing (or decreasing) the amount of a solid or liquid does not generate an equivalent change in the vaporous components (10 g does *not* indeed yield a 10 times increase in odor over 1 g). This is because the amount of odorant emitted from a given substance is also related to the substance's vapor pressure, the rate of evaporation or sublimation of the odorant(s), the total available surface area, and environment factors, such as ambient temperature, humidity, and air movement (99–101). Although operational and scientific communities frequently overlook the effect of surface area, altering surface area is a highly efficient way of altering odor availability in both testing and training scenarios. An odorant can only be released into the gaseous phase from the outer surfaces of a material, whether a solid or a liquid. For instance, a single square of C-4, a plastic explosive, will have less surface area and thus less odor availability than the same mass of C-4 spread out in a thin layer or cut up into many smaller cubes. Container opening size will have a similar effect—for a given volume of liquid, more odor will be available from a container or opening with a larger diameter. Thus, filling a container to the top is not necessarily an effective way to increase the amount of odor. Although, in an open container, increasing the size of the mouth or opening is indeed an effective way of increasing odor availability for the same volume of material, where a pin-sized hole will release a very low amount of odor compared to an open wide-mouthed jar. This can be an effective way of increasing or decreasing odor availability during testing. Likewise, in a closed container, once the headspace above the sample in the container is saturated with odor (i.e., equilibrium has been reached), a further increase in amount of material will not result in a greater concentration of odor (98, 102). For example, researchers placed 10 mg of triacetone triperoxide (TATP) in the bottom of a vial, and the crystals only covered ~10% of the bottom of the vial. The resulting vapor concentration from the vial was measured to be 80 ng/L at equilibrium. When 200 mg of TATP was placed in the vial, now covering 100% of the bottom, the resulting vapor concentration at equilibrium doubled to 160 ng/L. Finally, when the amount of material was further increased to 1,000 mg, which just increased the *volume* of TATP but not the *surface area* (still 100% coverage), the vapor concentration only increased by 18% to 190 ng/L (103).

There are many ways of characterizing and quantitating the odor profile of a given substance. The most common technique for measuring trace vapor components in the headspace is by solid phase microextraction (SPME) to extract the vapor molecules, paired with analysis by gas chromatography/mass spectrometry (GC/MS) for analysis of the extractant (104, 105). Unless a rigorous quantification method is used, which can be particularly arduous in SPME-GC/MS, each step in the analysis lends some amount of bias in the ratio of analytes measured. Meaning, the SPME fiber adsorbs some analytes preferentially to others, and the resulting data will yield a greater abundance of those analytes compared to others that may be present in the sample headspace in the same quantity. The gas chromatography column and mass spectrometer will also influence the ratio of analytes in the resulting data. It is thus important for researchers to understand that, with this or other headspace analysis methods, the ratios of measured odorants are not necessarily entirely reflective of the ratio that exists in nature.

Furthermore, the compounds that are in the highest abundance in the headspace, as determined by instrumental analysis, are not necessarily the same compounds that are *perceived* as having the greatest impact by dogs (106). Returning again to the cocaine example, Furton et al. (88) examined the headspace of multiple cocaine samples and found a number of volatile compounds present, to include methyl benzoate. Though methyl benzoate was not the dominant volatile species in the headspace, it was shown to be the active odorant of cocaine (88). Rice and Koziel (106) highlight that this discrepancy between what is measured instrumentally and what is perceived by the olfactory system has important implications in the creation and testing of mimic or surrogate training aids (106). The researchers compared instrumentally measured odorants from illicit drug samples and surrogate training aids to reported perceived olfactory intensity using both human and canine subjects. The results demonstrated that there was not a direct relationship between odorant concentration and perceived odor intensity, and that surrogates made using the compounds dominant in the instrumentally-determined odor profile, and *not* the *perceived* active odorants, would not elicit the same response.

### Odor Delivery

In the specific instance of olfactory detection threshold (ODT) testing, it is particularly important to maintain a known and constant source of odor at a given concentration throughout testing, and be able to deliver that odor at adjustable and accurate concentrations as the testing requires. This task can be quite challenging as evidenced by the high variability in published values of ODTs for dogs even when evaluating the same odorant (7, 8, 107). Factors such as previous training and familiarity with the odorant, individual differences between dogs, and testing protocols are potential sources of variation; however, differences in odor delivery methods are large contributors to such discrepancies (106). The two greatest factors are ab/dsorption to surrounding surfaces and dilution as odorants move away from the source. Whenever an odor source is contained or passes through a material, such as tubing in an olfactometer, some amount of the odorant is potentially lost due to ab/dsorption resulting in the delivery of a vapor concentration lower than intended. Though some amount of loss is likely to all materials, when delivering odor with an olfactometer or the like it is recommended to use Teflon or passivated materials (such as coated stainless steel) for all tubing through which the odor passes, and it is additionally recommended to heat these materials and to remove all possible cold spots from the airflow pathways to minimize losses to adsorption. If the odorant being tested has a high vapor pressure, these means should alleviate the majority of adsorption to the wetted portions of the flow path. Should the material being tested be of higher molecular weight/lower vapor pressure, quantitative measurements of the vapor concentration should be conducted to account for loss to adsorption and calculate the final concentration delivered. Finally, as soon as the vapor exits the port of an olfactometer or diffuses into the environment beyond the odor source or containment, the vapor plume or stream is diluted by surrounding air. Furthermore, air flow in the testing location may carry the odorants away from source further diluting the concentration. Designing the experiment in such a way that the dog has to place its muzzle into a portal or deep container with a smaller opening and ensuring the dogs are trained to bring their muzzle close to source will begin to alleviate this issue. Again, using quantitative measurements of the vapor concentration at the point where the dog samples is the best way to confirm the dog is experiencing the intended odor concentration (108).

### Contamination

Contamination and storage of target and non-target materials are essential and often inter-related considerations in maintaining the integrity of canine olfactory detection research. Contamination occurs when odor or scent is inadvertently transferred between materials or odor sources. A major source of contamination is the introduction of human scent to a target material. Mishandling targets can cause human scent to become associated with a given target, either confusing the odor profile or providing a secondary odor that dogs may learn to identify instead of the target odor. Further, scent trails of the people placing targets can contaminate testing areas, and provide dogs with a trail to follow toward a hidden target material (109–111). Contamination form saliva deposited on a target location can also provide inadvertent odor cues, which can occur when carousel setups are used if the positions are rotated but containers are not replaced (37, 112). As discussed above, the use of controls is important for minimizing the risk of dogs learning to respond to contaminating odorants rather than the target odor itself.

Cross-contamination occurs when the odor of one target is unintentionally transferred to another target, which can have varying effects on olfactory tests. For instance, dogs may incorrectly learn the target odor as a mixture of the contaminating odor and the target odor, and may fail to identify the pure trained material in a testing scenario. Cross-contamination most commonly occurs when different target materials are stored in close proximity to one another, otherwise known as “unit scent,” and is most prevalent when those materials have a large disparity in vapor pressure. For example, Hallowell et al. (113) found that likely cross-contamination of explosives stored together led to a preventable fault in canine training (113). The dogs were only able to detect compounds with the highest vapor pressures, and could not identify lower vapor pressure explosives that had been co-stored.

In a study of cross-contamination between co-stored training materials (birch, clove, and anise essential oils), the relative amounts of cross-contamination apparent were compared for three types of containment (114). In this experiment, 5 μL of each oil was placed on separate cotton swabs, stored inside one of three common primary containment systems (20 mL glass vials, 4 oz canning jars, or Mylar bags), and placed within a single outer jar. Cross-contamination, monitored over a 24-week period, was noted as early as week 1. Methyl salicylate, a volatile component of birch oil, was identified in the clove and anise samples of each primary containment system. Such cross-contamination between segregated materials has the potential to alter the odor profiles of target aids and affect the integrity of testing materials. Proper handling and storage of testing materials including the use of both primary and secondary containment can be very important as barriers for odor containment and protection of target materials, especially when materials must be stored in close proximity with other testing materials. The primary, or inner layer of containment, should not impart odor to the training material or react with it. A non-corrosive metal or glass containment is suggested for this layer, as plastics emit chemicals that can cause contamination. The secondary, or outer, containment should be a non-permeable material with a lid that eliminates leakage (72).

Another source of contamination results from residual odor, sometimes referred to as inverse contamination or contamination of the working environment, which occurs when the target material leaves remnants or volatiles in the environment where it was placed. This can often occur when a substance is left in direct contact with a surface, such as a table or drawer, and when the substance is allowed a period of time to sit before the testing session begins (57). Secondary transfer of odor can occur when odor from one material is transferred onto a surface, and then from that surface onto a second container. This is likely to occur when a target material is removed from a location in the testing scenario and a second material is placed on top of existing residual odor. A similar effect can be seen from transfer by touch when the individual preparing the test touches one testing material, contaminating the set of gloves, and then touches another testing material with the same gloves. Papet (115) and the UK Centre for the Protection of National Infrastructure each specifically warn against these risks, and suggest placing target materials on often-replaced barriers such as wax paper and changing gloves frequently to help limit residual odors (72, 115). Such contact contamination is, however, even more complicated. For example, a probable solution to such contact contamination is having separate individuals emplace target and non-target odor samples, but this strategy may result in the availability of a discriminable difference between the odor of the two individuals being associated with the different samples. This may happen regardless of the wearing and regular changing of gloves as canine olfaction is sensitive enough to detect the effluent from individuals that contaminates samples by just the individuals being in close proximity to the samples.

The duration of residual odors depends on the testing material itself, the substrate being contaminated by the residual odor, the amount of contamination, and environmental conditions. For example, residues from narcotics or essential oil have been shown to be detected anywhere from 2 to 48 h after removal of the odor source (12, 114, 116). Dogs have been able to detect human remains residue in soil up to 667 days after removal (117), and have been successful in locating blood on cotton swatches after five laundry cycles (118). Since residual odor can be difficult to predict, it is best to keep records of past testing odor locations to help identify apparent false alerts that are actually correct but caused by residual odor (72).

### Effects of Wrapping/Containment

It is nearly impossible to present a target substance free of any type of container or packaging. Particularly in an operational setting, the target of interest is likely to be securely wrapped, packaged, and/or obscured in some manner. Even in this situation, odorants from the target are likely to be present on the outer barrier for a number of reasons. This form of contamination can be problematic if the goal is to assess dogs' ability to detect odor that is concealed. The durability of odorants on the outside of a container is dependent on the amount and manner deposited and the tendency for the outer material to absorb the odorant in question. The rate of diffusion or permeation of odorants through the wrapping or packaging material is also dependent on the material type and thickness.

In a testing situation, such as olfactory threshold measurements, it is important to keep in mind that all packaging and wrapping around a target material will absorb some amount of the odor, even in “non-stick” materials such as Teflon. Using TNT vapors pulsed at various surfaces, Poziomek et al. (119) demonstrated that the TNT adsorbed more strongly to some surfaces tested than others, and, in fact, Teflon was the optimal substrate for adsorption, retention, and recovery of TNT (119). Again, the molecular structure of the odorant and type of wrapping, as well as temperature and other environmental factors, will affect ab/dsorption, and like with permeation, ab/dsorption can change the ratios of odorants in the odor profile with certain odorants being retained more strongly than others. For example, when odor profiles from living and deceased people were collected onto a sorbent material, it was shown that the resulting instrumentally measured odor profiles were dependent on the type of adsorbent material used in collection (120, 121).

Similar to wrapping, buried odor behaves and is transported to the surface for detection through complex processes. A body of literature has been devoted to describing buried odor, particularly in the case of landmine (122–128) and cadaver detection (117, 129, 130). As an overview, the evolution of buried odor involves dynamic processes of absorption, diffusion, dissolution in water, transformation by microbes, and uptake by vegetation that change with changing conditions. A detailed discussion is beyond the scope of this review, but in summary, for a dog to detect buried odor, free odor molecules must diffuse through soil to the surface. However, free odorant molecules may absorb to soil particles or dissolve into water, where they then may be carried away with ground water or taken up by the roots from nearby vegetation. This is the reason handlers often report of dogs not indicating a buried hide at source, but instead at a nearby water source or tree. The movement of free odorants is dependent on the type of odorant, the soil type, porosity, and moisture content, and the temperature, thermal radiation from sunlight, and air movement above the burial. In general, as soil becomes dry, more odor molecules absorb to the soil particles, lowering the odor availability. Moisture in the water enhances diffusion and increases odor availability (122, 128, 131). Because of the multifaceted nature of buried odor movement and availability, constructing reproducible testing with known variables is challenging. As such, any testing conducted with buried odor should be carried out with great care with as many defined variables as possible.

### Set Time

Allowing each dog to experience the odor in the same way each time requires the ability to confidently deliver a known and constant odor profile and odor concentration over the duration of a test or set of olfactory experiments. In order for the first and last dogs being tested (and all in between) to have access to the same concentration of odor, the sample must be delivered following a proper equilibration time for the chosen container and material being tested, commonly referred to as “set” or “soak” time in an operational or field setting. Unfortunately, there is no single equilibration time that is appropriate for all scenarios, but understanding the factors that affect equilibration time can assist researchers in making an educated decision given a particular set of experimental parameters. Many of the factors discussed above will affect soak time. In general, the higher the vapor pressure of the odorant of interest, the faster the system will come to equilibrium. The actual time will also be dependent on and change with the amount of material being used, the size and type of container, whether or not the odorant(s) must permeate through any sort of concealment, ambient temperature and humidity, air flow in the environment, and the presence and quantity of multiple odorants in the container. There are additional nuances to this, of course. For one instance, if the odorants do not simply evaporate/sublimate from the testing material, but instead evolve from a reaction of some sort, such as a decomposition reaction, time to equilibrium will also be dependent on the rate of that reaction. For instance, the explosive hexamethylene-triperoxide-diamine (HMTD) itself has a very low vapor pressure yielding very little molecular HMTD available as an odorant; however, it degrades under normal ambient conditions producing a number of detectable odorants, meaning equilibrium is dependent on the rate of the decomposition reaction (132).

Once an odorant has reached equilibrium in its container, the odor concentration will stay constant, assuming that none of the variables above change. However, this is not always the case. In one example, the odor profile associated with certain types of aluminum powder, a component of some homemade explosives, is derived from the breakdown of the stearic acid coating yielding a mix of odorants that, to humans, smell similar to crayons. Field measurements of the headspace of the aluminum powder on a cool morning yielded an abundance of odorants related to stearic acid decomposition, but when tested again later in the day on a warm afternoon the same amount of material yielded only very low levels of odorants. Further research indicated that exposure to heat generated by the sun on the warm afternoon actually drove off the odorants faster than they were produced from the stearic acid reaction (133). Though this describes a very unique set of circumstances and materials, it illustrated why it is important to consider not only the time required for equilibration, but also the duration the odor remains available. Depending on the source of the odor and the amount of substance being used, it is possible to deplete the available odorants over the duration of a lengthy test. Some commercially available training aids, for instance, have a short reported service-life of only several hours. In order to conduct a test that is reproducible and stable over its duration, it is thus imperative to be aware both when the substances being tested have reached equilibrium and when the odor begins to be depleted. The soak or set times selected by various canine certifying bodies are generally non-specific with many requiring a set time of at least 30 min with no maximum set time given (53, 134).

## Conclusions

A lack of standardization in canine olfactory detection assessments, both in scientific research and in evaluations of operational canines, has led to a wide variability in results. This lack of standardization partially stems from the wide range of aspects examined by olfactory detection research. Nonetheless, attempts should be made to increase consistency in methodologies, such as standards for necessary controls to include and reporting of data, to allow for ease of interpreting results, internal validity of data, and making meaningful comparisons across studies. In this review, we discuss the range of factors that should be considered when designing and conducting canine olfactory detection studies, many of which have direct applications to operational testing.

It is important for researchers to conduct both basic and applied research related to canine detection. However, it should be cautioned that not all research can be extrapolated to operational performance due to variables discussed in this review. Specific variables influence the quantification of detection dog performance such as experimental design, testing bias, odor contamination, training aid storage/handling, odor characteristics, experimental controls, and statistical analysis. Methods for testing canine scent detection vary influencing the outcome metrics of performance and the validity of results. Operators, management teams, policy makers, and law enforcement rely on scientific data to make decisions, design policies, and to advance canine technologies. Therefore, scientists conducting research should incorporate as many operational constraints as possible so that the data can be applied to operational performance. In addition, operational teams should adopt rigorous scientific standards in order to scientifically validate their dogs' capabilities. This will lead to better informed decisions about capability, vulnerability, and risk analysis.

## Author Contributions

LL, SK, LD, and AS wrote the first draft of the manuscript. CA, LW, and MS made significant revisions and contributions to the content. All authors read and approved the final draft.

## Conflict of Interest

AS was employed by the company AGS Forensics, LLC. The remaining authors declare that the research was conducted in the absence of any commercial or financial relationships that could be construed as a potential conflict of interest.
